# Reducing dynamical electron scattering reveals hydrogen atoms

**DOI:** 10.1107/S2053273318013918

**Published:** 2019-01-01

**Authors:** Max T. B. Clabbers, Tim Gruene, Eric van Genderen, Jan Pieter Abrahams

**Affiliations:** a Center for Cellular Imaging and NanoAnalytics (C-CINA), Biozentrum, University of Basel, Mattenstrasse 26, CH-4058 Basel, Switzerland; b Paul Scherrer Institut (PSI), CH-5232 Villigen PSI, Switzerland; c Leiden Institute of Biology, Sylviusweg 72, 2333 BE Leiden, The Netherlands

**Keywords:** dynamical scattering, electron diffraction, hydrogen atoms, nanocrystals, hybrid pixel detector

## Abstract

Compared with X-rays, electron diffraction faces a crucial challenge: dynamical electron scattering compromises structure solution and its effects can only be modelled in specific cases. Dynamical scattering can be reduced experimentally by decreasing crystal size but not without a penalty, as it also reduces the overall diffracted intensity. In this article it is shown that nanometre-sized crystals from organic pharmaceuticals allow positional refinement of the hydrogen atoms, even whilst ignoring the effects of dynamical scattering during refinement. To boost the very weak diffraction data, a highly sensitive hybrid pixel detector was employed. A general likelihood-based computational approach was also introduced for further reducing the adverse effects of dynamic scattering, which significantly improved model accuracy, even for protein crystal data at substantially lower resolution.

## Abstract 2

Experimental and computational reduction of dynamical electron scattering allows for visualizing of individual hydrogen atoms.

## 1. Introduction   

Electron crystallography allows for the solving of structures of beam-sensitive macromolecules and organic compounds using sub-micron-sized three-dimensional crystals. The minute sample volumes used in electron diffraction are limiting the maximum radiation dose and the diffracted intensity (Henderson, 1995 ▸). Electrons interact with matter so strongly that the majority of 300 keV electrons will have scattered either elastically or inelastically after having passed through only 50 nm of organic matter (Clabbers & Abrahams, 2018 ▸). Because this was considered to prevent successful analysis of three-dimensional protein crystals, macromolecular electron crystallography was limited until recently to two-dimensional crystals (Unwin & Henderson, 1975 ▸; Gonen *et al.*, 2005 ▸). Chemical electron crystallography using inorganic and organic crystals developed independently from protein crystallography (Cowley, 1953*a* ▸,*b* ▸; Vainshtein, 1964 ▸; Dorset, 1995 ▸; Kolb *et al.*, 2007 ▸; Mugnaioli *et al.*, 2009 ▸; Zhang *et al.*, 2010 ▸; Zou *et al.*, 2011 ▸). Recently, the rotation method that has been the standard for decades in X-ray protein crystallography (Arndt & Wonacott, 1977 ▸; Dauter, 1999 ▸) was adapted to electron crystallography for determining the structure of beam-sensitive macromolecules (Nederlof *et al.*, 2013 ▸; Nannenga, Shi, Leslie *et al.*, 2014 ▸; Nannenga, Shi, Hattne *et al.*, 2014 ▸; Yonekura *et al.*, 2015 ▸; Clabbers *et al.*, 2017 ▸), organics and inorganics (Gemmi *et al.*, 2015 ▸; van Genderen *et al.*, 2016 ▸; Gruene *et al.*, 2018 ▸).

Analogous to similar developments in X-ray crystallography and single-particle cryo-EM (Broennimann *et al.*, 2006 ▸; Kühlbrandt, 2014 ▸; McMullan *et al.*, 2016 ▸), the introduction of new camera technologies, such as the advent of hybrid pixel detectors, was of vital importance for the development of electron diffraction of beam-sensitive crystals, as they offer high sensitivity, fast read out and reduced background (van Genderen *et al.*, 2016 ▸; Tinti *et al.*, 2018 ▸). Electron diffraction data integration is feasible with existing X-ray crystallography packages, including profile fitting and scaling (Leslie, 1999 ▸; Kabsch, 2010 ▸; Winter *et al.*, 2018 ▸), and requires only minor adaptations (Clabbers *et al.*, 2018 ▸). The introduction of electron counting detectors required a Poisson error model and background estimation optimized for weak data with low-background, which were implemented for integrating very weak, few-photon, X-ray diffraction data as measured by hybrid pixel detectors (Kabsch, 2010 ▸; Parkhurst *et al.*, 2016 ▸).

In general, crystallographic structure determination assumes single, kinematical scattering. Given the strong interaction of electrons with matter, multiple elastic or dynamical scattering is a major concern in electron crystallography, as it changes the observed structure-factor amplitudes (Cowley & Moodie, 1957 ▸; Glaeser & Downing, 1993 ▸; Dorset, 1995 ▸; Weirich *et al.*, 2000 ▸). Dynamical scattering cannot be removed by zero-loss energy filtering, and coincides with the kinematic Bragg scattering angles. On average, dynamical scattering increases the intensity of weaker reflections, whereas the strong reflections become less intense. Typically, dynamical scattering out of intense Bragg reflections into the weaker ones predominantly affects adjacent reflections when low-resolution terms dominate (Weirich *et al.*, 2000 ▸). Thus, it depends on the diffraction geometry and can therefore be reduced by averaging corresponding intensities obtained from multiple crystal orientations. This explains why precessing or tilting the electron beam and/or rotating the crystal in a random orientation, combined with averaging symmetry-related reflections, reduces the effects of dynamical scattering (*e.g.* Vincent & Midgley, 1994 ▸).

Crystal size is an important factor affecting dynamical scattering as the probability of multiple scattering events increases with sample thickness (Subramanian *et al.*, 2015 ▸; Clabbers & Abrahams, 2018 ▸). A small crystal size combined with data acquisition using the rotation (or precession) method reduces dynamical scattering. This approach allows for full integration of the Bragg reflections resulting in a better estimate of the kinematic scattering intensity (Arndt & Wonacott, 1977 ▸; Dauter, 1999 ▸; Vincent & Midgley, 1994 ▸). It benefits from an electron-counting quantum area detector with a high dynamic range to optimize the weak diffraction signal. Dynamical refinement of electron diffraction data is an effective, independent approach for dealing with dynamical diffraction (Jansen *et al.*, 1998 ▸; Palatinus, Petříček & Corrêa, 2015 ▸; Palatinus, Corrêa *et al.*, 2015 ▸; Palatinus *et al.*, 2017 ▸). It requires knowledge of the atomic crystal structure, the crystal shape or thickness, and the three-dimensional orientation of the crystal for each unmerged reflection. Because it models the electron wavefunction travelling through the entire crystal, dynamical refinement is computationally demanding and has so far only been implemented for relatively straightforward cases with small unit cells.

A potential benefit of electron diffraction is the improved contrast of hydrogen atoms. For the lighter elements (up to about sulfur) the atomic scattering cross sections are approximately proportional to 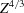, instead of 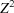 as for X-ray diffraction (Egerton, 2011 ▸). The increased contrast of light atoms relative to heavier ones implies a larger contribution from the hydrogen atoms to the overall signal (Cowley, 1953*a* ▸,*b* ▸; Vainshtein, 1964 ▸; Dorset, 1995 ▸; Clabbers & Abrahams, 2018 ▸). Localizing hydrogen atoms can be notoriously difficult in X-ray crystallography, even with high-resolution data. However, recently, hydrogen positions in organic and inorganic samples could be refined by reducing the effects of dynamical scattering by combining precession electron diffraction with dynamical refinement (Palatinus *et al.*, 2017 ▸).

Here, we present electron diffraction data for three-dimensional nanocrystals of two pharmaceutical organic compounds, recorded at cryogenic temperature using the rotation method and a Timepix hybrid pixel detector. The small size of the crystals reduced dynamical scattering, whilst the highly sensitive hybrid pixel detector boosted the weak diffraction signal. This combination allowed for localizing of the positions of individual hydrogen atoms at a very early stage of the refinement, and allowed unconstrained refinement of the hydrogen atoms, without any modelling of dynamical scattering. Furthermore, we introduce a likelihood-based approach for correcting dynamical scattering that down-weights overestimated reflection intensities as a function of intensity and resolution, in an approach similar to Wiener filtering. These corrections significantly improved the quality of the data and the model accuracy, and even work for protein crystal data with a substantially lower resolution.

## 2. Methods   

### 2.1. Data acquisition   

Diffraction data were acquired for two pharmaceutical organic compounds (experimental data are available online at https://doi.org/10.5281/zenodo.1407682) (see Fig. S1 in the supporting information), kindly provided by Novartis, corresponding to CCDC entries IRELOH (C_16_O_5_H_18_) (Dai *et al.*, 2010 ▸) and EPICZA (C_18_O_6_N_2_S_2_H_16_) (Deffieu *et al.*, 1977 ▸) in the Cambridge Structural Database (CSD). Data were acquired by continuous rotation at cryogenic temperature with an ∼2.0 µm-diameter parallel beam using an FEI Polara TEM, operated at 200 kV and equipped with a 512 × 512 pixel Timepix hybrid pixel detector (van Genderen *et al.*, 2016 ▸; Gruene *et al.*, 2018 ▸). The effective detector distance was determined with an aluminium powder standard (Ted Pella).

### 2.2. Data processing   

The raw data recorded from the detector were corrected for the pixel offsets between individual chips and converted to CBF (crystallographic binary file) format (see https://strucbio.biologie.uni-konstanz.de/xdswiki/index.php/Timepix2cbf; Gruene *et al.*, 2018 ▸). Data were integrated and scaled using *XDS* (Kabsch, 2010 ▸). The Laue group was constrained to *mmm*. Multiple crystal data sets were merged, scaled and converted to *SHELX* format within *XDS* (Kabsch, 2010 ▸). Data were truncated where there was still significant correlation at approximately *I*/σ(*I*) > 1.0 and *CC*_1/2_ > 50% (Karplus & Diederichs, 2012 ▸; Diederichs & Karplus, 2013 ▸).

### 2.3. Structure determination   

#### 2.3.1. Structure solution and model refinement   

Structures of both organic compounds were solved using *SHELXT* with default settings (Sheldrick, 2015*b* ▸). The models were refined using *SHELXL* (Sheldrick, 2015*a* ▸) and built in *SHELXLE* (Hübschle *et al.*, 2011 ▸). Hydrogen atoms were placed automatically when possible using HFIX. For anisotropic refinement against incomplete data, mild restraints were applied in *SHELXL* to ensure similarity (RIGU) (Thorn *et al.*, 2012 ▸) and positivity (XNPD 0.001) of the atomic displacement parameters. Electron atomic scattering factors were fitted to the nine Cromer–Mann coefficients as used by *SHELXL* (see also Fig. S11 in the supporting information) (Peng, 1999 ▸; Gruene *et al.*, 2018 ▸).

#### 2.3.2. Lattice parameter refinement   

In electron diffraction, the unit-cell dimensions and sample to detector distance are highly correlated because of the short electron wavelength. Therefore, both cannot be refined reliably at the same time from the diffraction data alone. However, the lattice can also be refined independently from the detector distance by minimizing the deviation from ideal model geometry (Gruene *et al.*, 2018 ▸). Geometrical restraints on bond lengths (DFIX) and bond angles (DANG) for IRELOH and EPICZA were generated using the *GRADE* server (http://grade.globalphasing.org/cgi-bin/grade/server.cgi) (see Tables S9 and S10 in the supporting information). The unit-cell parameters were then refined against the idealized interatomic distances using *SHELXL* and the program *CellOpt* (https://github.com/JLuebben/CellOpt). The geometrical restraints were only used for lattice refinement. After the geometrical restraints were removed from the instruction file, the model was refined in *SHELXL* against the new unit-cell parameters.

#### 2.3.3. Refining hydrogen positions   

To illustrate the quality of the data and the stability of the hydrogen positions, we refined their positions by subsequently removing the constraints on the hydrogen atoms (AFIX), which are normally refined using the riding model in *SHELX*. The hydrogen positions were then refined isotropically in *SHELXL* until convergence.

#### 2.3.4. Validation   

The models were validated using *R*_complete_ (Luebben & Gruene, 2015 ▸), as a more robust alterative to *R*_free_ (Brünger, 1997 ▸). Since all reflections are used to calculate *R*_complete_, the model can be refined against all data; hence *R*_work_ is equal to *R*1. The *R*_complete_ was calculated with a test set size of 0.2%, running ten refinement cycles for each run, using the *R_complete* graphical user interface (https://github.com/JLuebben/R_complete).

To compare the quality of the model geometry, we calculated the r.m.s.d. of the electron diffraction structure bond lengths from the reference X-ray models (Deffieu *et al.*, 1977 ▸; Dai *et al.*, 2010 ▸). We assessed the stability of the refined hydrogen atoms by calculating the r.m.s.d. of the hydrogen-bond lengths (*X*—H) compared with the idealized hydrogen-bond-length tables generated by *SHELXL* using the NEUT command (Sheldrick, 2015*a* ▸). Using NEUT will list the inter-nuclei distances as neutron scattering occurs on the nuclei, and since idealized hydrogen-bond lengths determined by X-ray diffraction are generally too short, it is therefore appropriate to use the more accurate *X*—H distances for neutron diffraction as described previously (Gruene *et al.*, 2014 ▸).

### 2.4. Dynamical scattering corrections   

#### 2.4.1. Dynamical scattering increases intensity of weaker reflections   

We previously observed an overestimation of the weaker reflection intensities in our electron diffraction data (van Genderen *et al.*, 2016 ▸; Clabbers *et al.*, 2017 ▸). We confirmed that this was also the case for the two organic samples presented here by plotting the observed structure-factor amplitudes 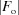 against the structure-factor amplitudes calculated from the model 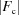 (Figs. 3 and 4). On average, dynamical scattering increases the intensity of weaker reflections at the expense of the strong reflections, so we can expect the observed structure-factor amplitudes 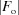 to be overestimated for the weaker reflections (Dorset, 1995 ▸; Weirich *et al.*, 2000 ▸; Subramanian *et al.*, 2015 ▸; Clabbers & Abrahams, 2018 ▸). Assuming that we have a complex-valued dynamical scattering component 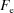, which is uncorrelated to the kinematical structure factor 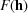, we can describe the expected value of 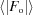 using a hyperbolic function defined as
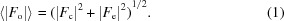
Using least-squares fitting, we can derive the expected dynamical error term 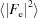 over all observations, where the hyperbolic function describing 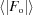 intercepts the *y* axis at 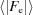 (Figs. 3 and 4).

We determined 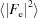 as a function of resolution by dividing the data into ten equally sized resolution bins (in terms of number of reflections), and within each bin we determined the corresponding resolution-dependent dynamical scattering error 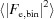 using least-squares fitting. Using the curve-fitting tool in *MATLAB* we established the relation between 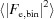 and the resolution *d* (Figs. 3*c* and 4*c*), resulting in the continuous function 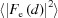.

In the absence of dynamical scattering (when 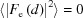), the measured and calculated structure factors should be linearly correlated. Thus, as a function of resolution *d*, we can infer the scale 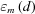 by which an expected observed intensity 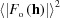 is increased by dynamical scattering, as a function of (i) the resolution *d*, (ii) the strength of the dynamic effect 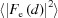, and (iii) the intensity 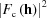 that would be observed in the absence of errors, 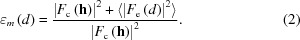
Thus, 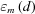 is essentially equivalent to a reciprocal generalized Wiener filter [see equation (10) in Pratt (1972 ▸)].

#### 2.4.2. Dynamical scattering corrections for high-resolution data   

Refinement in *SHELX* uses scaled observed intensities, which are assumed to be kinematic, *i.e.* 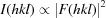. Instead of refining against the measured intensities, we refined against corrected intensities 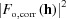 according to 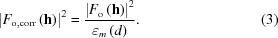
These corrections were applied to each reflection, on both the intensities and their sigma values, using a single line of Awk commands that also writes the corrected HKL file with the standard formatting required for *SHELX*. The model was refined in *SHELXL* against the corrected data. The positional accuracies of the hydrogen atoms were assessed as before by refinement without any constraints on the hydrogen bonds.

#### 2.4.3. Dynamical scattering corrections for low-resolution protein diffraction data   

In a similar fashion, the likelihood-based corrections for dynamical scattering were applied to low-resolution data. Previously, we observed the same apparent overestimation of the weaker reflections for hen egg-white lysozyme nanocrystals, solved up to a resolution of 2.1 Å (PDB ID 5o4x; experimental data are available online at https://doi.org/10.5281/zenodo.1250447; Clabbers *et al.*, 2017 ▸). As protein data are generally refined against the amplitudes instead of intensities, we corrected structure factors according to
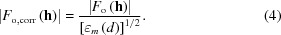
The resolution dependency of 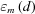 was determined from the dynamical scattering errors 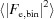 within ten equally sized resolution bins (in terms of number of reflections). Here a discrete correction was applied for each resolution bin independently, rather than fitting a multi-parameter continuous function to describe the observed curve of 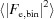 as a function of resolution *d* (Fig. 5*b*). In all cases, the models were refined until convergence.

## 3. Results   

### 3.1. Structure determination   

#### 3.1.1. IRELOH   

**Figure 1:**
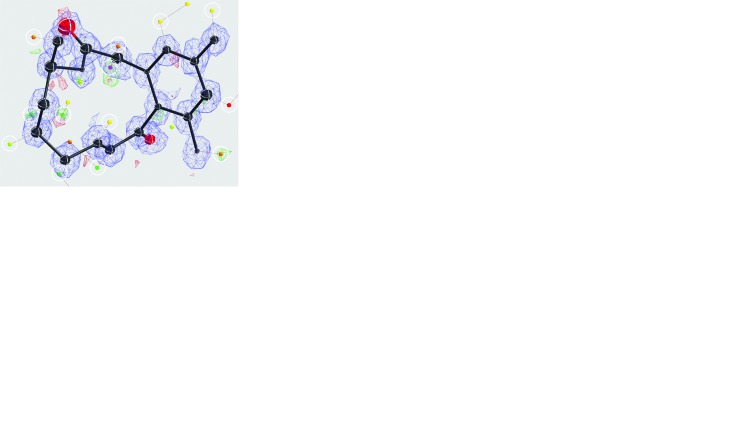
Structure determination of IRELOH. (*a*) The model after phasing using the direct methods program *SHELXT*. Small icosahedra represent maxima in the difference map. Peak heights are colour coded in rainbow colours (purple = high, red = low). Icosahedra that indicate the positions of individual hydrogen atoms are encircled. (*b*) Automated placement of the hydrogen atoms by *SHELXLE* confirms the quality of the model’s geometry, placing all hydrogen atoms correctly except for H5. (*c*) Final model after manual building and anisotropic refinement (RIGU, XNPD 0.001) in *SHELXL*. (*d*) Unconstrained positional refinement of the hydrogen atoms. (*e*) *ORTEP* plot with the numbering for the non-hydrogen atoms of the final model. Parts (*a*)–(*d*) were drawn using *SHELXLE* with default contour levels of 2.7σ for the 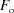 − 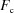 difference map and 1.2σ for the 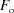 map. The atomic displacement ellipsoids are colour coded black for carbon and red for oxygen, while the hydrogen atoms are represented in white.

Diffraction data were acquired for three IRELOH nanocrystals over a combined tilt range of 144° using the rotation method (see Fig. S1 and Table S2 in the supporting information). Merging resulted in 85% completeness up to a resolution of 0.82 Å, which allowed phasing by direct methods (see Table 1 ▸ and Fig. S4 in the supporting information). The resulting model after phasing reveals the presence of individual hydrogen atoms as indicated by the difference map (Fig. 1 ▸ *a*).

Automated fixing of the hydrogen atoms can place all but one, indicating the quality of the model (Fig. 1 ▸ *b*). Although there was clear difference potential, position H5 was not placed automatically based on the model geometry and had to be set manually using AFIX 148 (Fig. 1 ▸ *b*). The final model after anisotropic refinement shows a well resolved map (Fig. 1 ▸ *c*) and high-quality model geometry (Fig. 1 ▸ *e*), although the standard crystallographic quality indications are rather poor (Table 2 ▸).

Removing the constraints on the 18 hydrogen atoms allowed refining of their positions and bond lengths despite increasing the number of free parameters from 196 to 245, with a total of 156 restraints (Table 3 ▸). The resulting structural model shows a remarkably high consistency of hydrogen atoms (Fig. 1 ▸ *d*).

#### 3.1.2. EPICZA   

**Figure 2:**
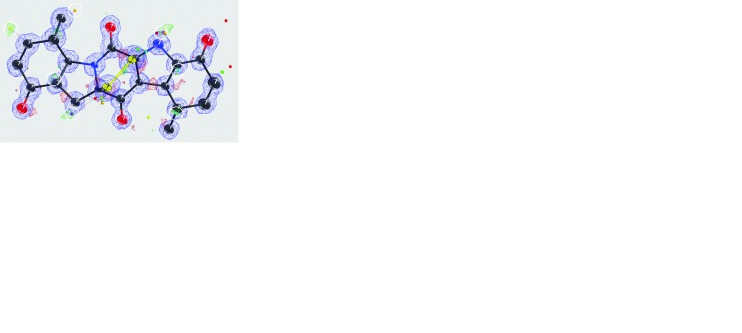
Structure determination of EPICZA. (*a*) The model after phasing using the direct methods program *SHELXT*. Small icosahedra represent maxima in the difference map. Peak heights are colour coded in rainbow colours (purple = high, red = low). Icosahedra that indicate the positions of individual hydrogen atoms are encircled. (*b*) Automated placement of the hydrogen atoms by *SHELXLE* showing the quality of the model’s geometry, placing all hydrogen atoms apart from H3, H5 and H5B. (*c*) Final model after building and anisotropic refinement (RIGU, XNPD 0.001) in *SHELXL*. (*d*) Unconstrained positional refinement of the hydrogen atoms where the positions of H7 and H10 are unstable and move out of bounds. (*e*) *ORTEP* plot with the numbering for the non-hydrogen atoms of the final model. Parts (*a*)–(*d*) were drawn using *SHELXLE* with default contour levels of 2.7σ for the 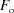 − 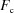 difference map and 1.2σ for the 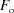 map. The atomic displacement ellipsoids are colour coded black for carbon, red for oxygen, blue for nitro­gen and yellow for sulfur, while the hydrogen atoms are represented in white.

Data were acquired for four EPICZA nanocrystals using the rotation method, with a total tilt range of 213° (see Fig. S1 and Table S3 in the supporting information). Merging yielded close to complete data up to a resolution of 0.83 Å (see Table 1 ▸ and Fig. S4 in the supporting information). Although the EPICZA molecule has twofold symmetry, this was not used or imposed for structure solution or refinement. Direct methods allowed for the calculating of a map revealing difference potential peaks for localizing individual hydrogen atoms (Fig. 2 ▸ *a*).

Most hydrogen atoms could be placed automatically, indicating good data quality (Fig. 2 ▸ *b*). Hydrogen atoms H3, H5, and H5B were placed with the coordinates of the corresponding difference peaks observed during refinement with AFIX 147, 23 and 147 (Fig. 2 ▸ *b*), respectively. After anisotropic refinement, the final model shows high quality of the map and accurate model geometry (Figs. 2 ▸ *c* and 2 ▸ *e*), but again with relatively poor quality indicators (Table 2 ▸).

In contrast to the X-ray model, we did not observe any density indicating the presence of water molecules. Perhaps this was because of evaporation after inserting the sample into the vacuum column. This would also explain the observed shrinkage of the unit cell by 90 Å^3^ compared with the X-ray model. Given the *P*2_1_2_1_2_1_ crystal symmetry, this shrinkage corresponds to a cube with sides of 2.83 Å per missing H_2_O molecule, which is very close to the volume of one H_2_O molecule in liquid water, which on average occupies a cube with sides of 3 Å.

All 16 hydrogen-atom positions and bond lengths were refined by removing the constraints, increasing the number of free parameters from 259 to 305, with a total of 267 restraints (Table 3 ▸). Almost all refined hydrogen-atom positions are stable, but H7 and H10 are unstable and move too far away to have a bonding interaction with the non-hydrogen atom (Fig. 2 ▸ *d*).

### 3.2. Dynamical scattering corrections   

#### 3.2.1. IRELOH   

**Figure 3:**
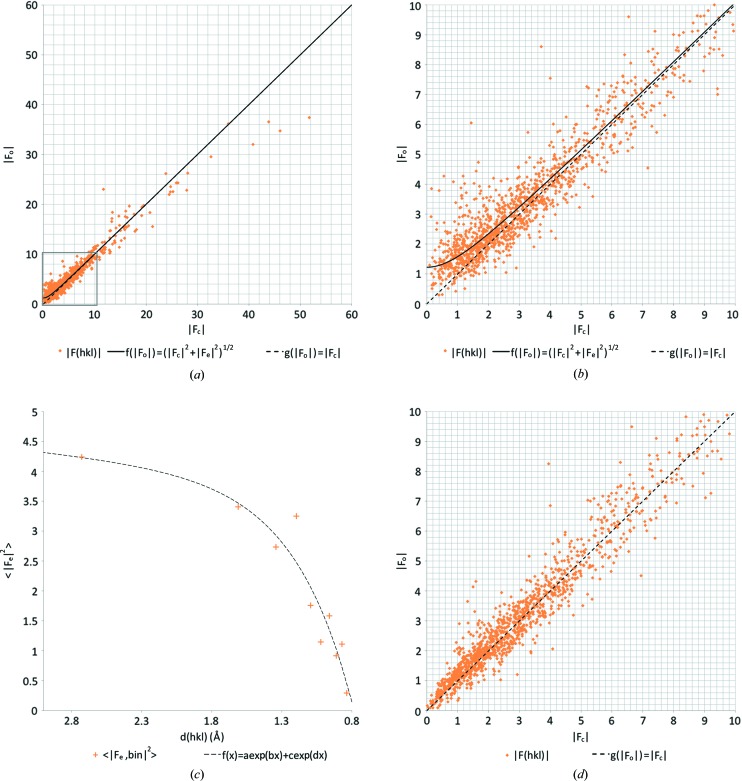
Likelihood-based corrections for dynamical scattering. (*a*) 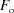 *versus* 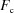 plot for IRELOH where 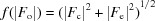 is a hyperbolic function with an expected dynamical scattering error term of 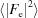 = 1.51 as determined by least-squares fitting, and where 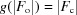 shows a perfect linear correlation. (*b*) Zoomed-in version on the lower intensity reflections, as marked with a grey box in (*a*). (*c*) 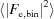 as a function of the resolution, where we fitted an exponential curve 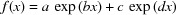 with parameters *a* = 3.64, *b* = 0.058, *c* = −24.67 and *d* = −2.38. (*d*) 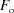 *versus* 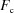 plot after applying the likelihood-based corrections shows an improved correlation between 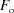 and 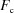 with an error of 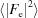 = 0.33.

We observed considerable dynamical scattering, leading to a clear overestimation of the lower intensities (Fig. 3 ▸ *a* and 3*b*). The overestimation decreased with increasing resolution. We fitted an exponential curve to the data, thus defining 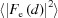 as a continuous function of the resolution (Fig. 3 ▸ *c*). This allowed likelihood-based corrections of the intensities dependent on both intensity and resolution using equation (3).

The likelihood-based corrections for dynamical scattering significantly improved the fit of the model to the data, with an *R*1 of 13.2% and an *R*_complete_ of 15.1% (Table 2 ▸). Furthermore, the gap between *R*1 and *R*_complete_ decreases after the likelihood-based corrections were applied, indicating reduced bias. Although the model geometry of non-hydrogen atoms was unaffected (see Table 2 ▸ and Table S5 in the supporting information), the r.m.s. deviations from the idealized hydrogen-bond lengths did improve considerably (see Table 3 ▸ and Table S6 in the supporting information). The improvement can also be inferred from the structure-factor plots that show an almost linear correlation when least-squares fitting the same hyperbolic curve (Fig. 3 ▸ *d*).

#### 3.2.2. EPICZA   

**Figure 4:**
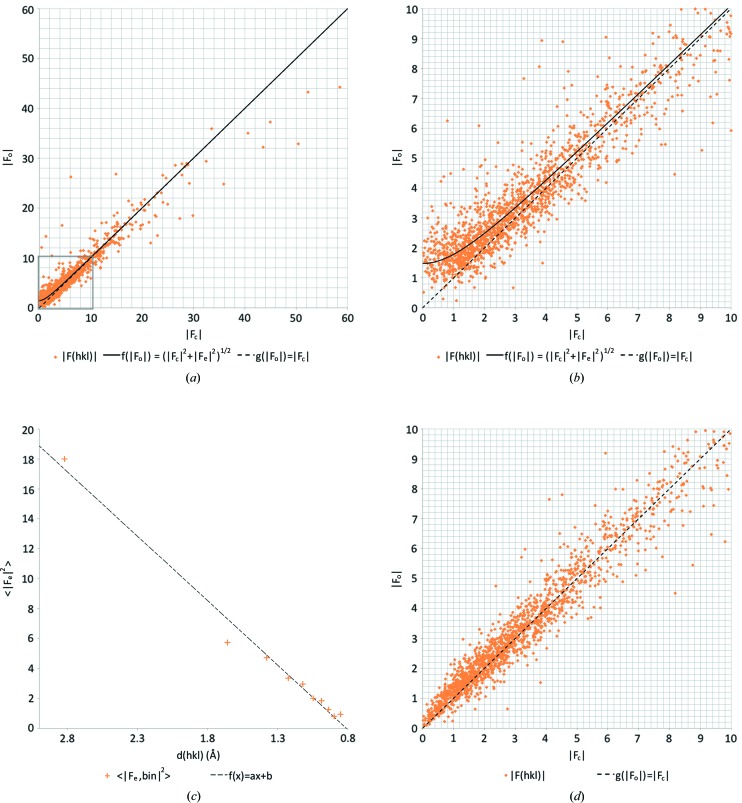
Likelihood-based corrections for dynamical scattering. (*a*) 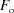 *versus* 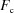 plot for EPICZA where 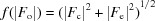 is a hyperbolic function with an expected dynamical scattering error term of 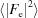 = 2.20 as determined by least-squares fitting, and where 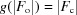 shows a perfect linear correlation. (*b*) Zoomed-in version of the same plot on the lower intensity reflections, as marked with a grey box in (*a*). (*c*) 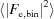 as a function of the resolution, where we fitted an linear model 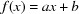 with parameters *a* = 8.61 and *b* = −6.98. (*d*) 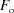 *versus* 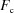 plot after applying the likelihood-based corrections shows an improved correlation between 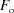 and 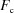 with an error of 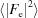 = 0.43.

Dynamical scattering affected the observed intensities of the structure factors (Figs. 4 ▸ *a* and 4*b*). Again, the dynamical scattering error 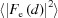 decreased with the resolution, and a linear curve was fitted to the resulting plot (Fig. 4 ▸ *c*). Using equation (3) we then made likelihood-based corrections of each reflection as a function of intensity and resolution.

The likelihood-based corrections improved the fit between the model and the experimental data, leading to a significant improvement in *R*1 and *R*_complete_ (Table 2 ▸). The model geometry also improved significantly. The r.m.s. deviations of bond lengths of the non-hydrogen atoms decreased (see Table 2 ▸ and Table S7 in the supporting information), as did the deviations from idealized hydrogen-bond lengths (see Table 3 ▸ and Table S8 in the supporting information). After applying the likelihood-based corrections, the 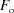 *versus* 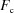 plot improved and also showed linear correlation for the weaker reflections with a much lower value for 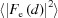 (Fig. 4 ▸ *d*).

#### 3.2.3. Lysozyme   

**Figure 5:**
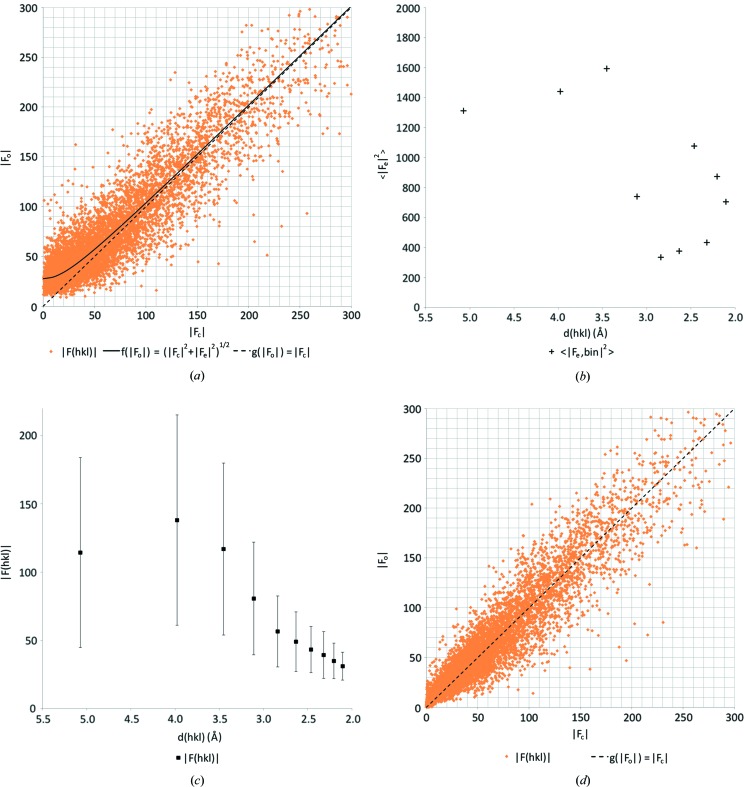
Likelihood-based corrections for dynamical scattering for low-resolution protein data. (*a*) 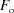 *versus* 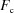 plot for hen egg-white lysozyme where 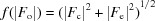 is a hyperbolic function with an expected dynamical scattering error term of 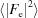 = 768.13 as determined by least-squares fitting, and where 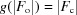 shows a perfect linear correlation. (*b*) 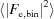 as function of resolution. (*c*) Mean structure-factor amplitude 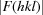 as a function of the resolution; the same resolution bins were used as in (*b*) and error bars indicate the standard deviation. (*d*) 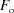 *versus* 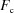 plot after applying discrete likelihood-based corrections, showing an improved correlation between 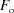 and 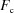 with an error of 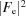 = 53.3.

The effect of dynamical scattering on the intensities had been observed previously from protein data at 2.1 Å (PDB ID 5o4x; experimental data are available online at https://doi.org/10.5281/zenodo.1250447; Clabbers *et al.*, 2017 ▸). Again, the weaker reflections in the 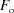 *versus* 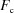 plot were overestimated (Fig. 5 ▸ *a*). We plotted the dynamical scattering error 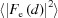 as a function of resolution, showing a non-linear relation (Fig. 5 ▸ *b*). We assume this non-linearity resulted from the presence of secondary structural elements and solvent contributions (Fig. 5 ▸ *c*). We made discrete likelihood-based corrections per resolution bin as described in Section 2.4.3.

The likelihood-based correction resulted in significant improvement of the model, as indicated by the reduction of *R*_complete_ from 29.1% to 26.2%. It also reduced model bias, as witnessed by the smaller gap between *R*1 and *R*_complete_. Furthermore, the 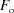 *versus* 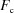 plot improves with a lower dynamical error value (Fig. 5 ▸ *d*). It is unclear why the average *B* factor increased upon correcting for dynamical scattering. However, the model geometry after applying the corrections showed a significant improvement, since r.m.s. deviations from ideal bond lengths and bond angles dropped by about 10% (Table 4 ▸).

## 4. Discussion and conclusions   

Electron diffraction allows structure solution even when only small crystals are available, and results in increased contrast of hydrogen atoms compared with X-ray diffraction. Here, we show that scattering potential at individual hydrogen-atom positions can be visualized after solving the structure by direct methods, even before interactive model improvement (Figs. 1 ▸ and 2 ▸). The refined coordinates are of comparable quality to the respective X-ray structures, indicating that organic structures can be solved with electron diffraction at sufficient quality to allow for further interpretation, for example for drug development by modelling. The positions of the individual hydrogen atoms are remarkably stable, allowing unconstrained refinement of the hydrogen-atom parameters.

Dynamical scattering was reduced experimentally by selecting for minimal crystal size, and by collecting rotation data from multiple crystals in random orientations. The signal-to-noise ratio was further boosted by measuring at cryogenic temperatures and using a highly sensitive hybrid pixel detector. Profile fitting of weak intensities allowed data to be extracted at or even below the noise level (French & Wilson, 1978 ▸; Oatley & French, 1982 ▸; Kabsch, 2010 ▸).

Acquiring more data to further increase multiplicity will always benefit data accuracy. Although it would allow a more accurate estimation of the kinematic intensity by averaging out orientation-dependent dynamical deviations, it cannot completely eliminate overestimation of weaker reflection intensities. Dynamical scattering effects could, in principle, also be further reduced by increasing the acceleration voltage, whilst data quality and structure refinement benefit from additional calibrations of the experiment (Gemmi *et al.*, 2015 ▸; Yonekura *et al.*, 2015 ▸), zero-loss energy filtering to remove inelastically scattered electrons (Yonekura *et al.*, 2002 ▸) and modelling of partial charge (Yonekura & Maki-Yonekura, 2016 ▸; Yonekura *et al.*, 2018 ▸). We consider these additional, independent measures to be important, but beyond the scope of our article, as they are enhanced, rather than replaced, by the methods we discuss here.

Existing methods of structure refinement that compensate for the effects of dynamical scattering rely on knowledge of the atomic crystal structure. Comprehensive modelling of dynamical scattering by either multi-slice or Bloch-wave simulations is computationally challenging. Current implementations assume perfect crystallinity and small unit cells with a limited number of atoms; increasing data multiplicity also increases the computational burden (Jansen *et al.*, 1998 ▸; Palatinus, Petříček *et al.*, 2015 ▸; Palatinus, Corrêa *et al.*, 2015 ▸; Palatinus *et al.*, 2017 ▸). There is currently no implementation for correcting protein data. Here, we introduced a likelihood-based approach, akin to the Wiener filter, that applies a straightforward scaling factor 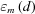 for down-weighting overestimated intensities as a function of intensity and resolution. It is computationally undemanding, has no underlying assumptions concerning crystal quality or thickness, and is sufficiently general to be implemented straightforwardly even for very complex cases. It is also sufficiently general to even allow corrections of single-particle cryo-EM data. We show it can substantially improve the fit between the model and experimental data, at the expense of only a few extra parameters, can reduce bias (as witnessed by a smaller difference between *R*1 and *R*_complete_), and has a positive effect on the model geometry (Table 2 ▸). In the absence of a predictive theory for the dependency of 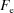 on *d*, the resolution dependency of 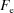 needs to be determined heuristically. For plate-like crystals or needle-shaped crystals that are rotated about an axis normal to their longest dimension, 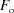 *versus* 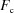 curves should also be checked as a function of rotation angle, as at higher angles the electron beam travels through the crystal for a longer distance. This implies stronger dynamical scattering. In theory, the relationship between 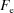 and 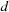 can be derived for each crystal by means of a full dynamical simulation. In that case it would be preferable to use the simulation results. Where dynamical calculations are not possible or practical, we therefore suggest determining 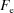 as a function of resolution by analysing 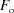 *versus* 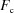 curves for different resolution bins.

Like the other approaches, the implementation of our method requires an initial model to calculate its structure-factor amplitudes. However, it has frequently been observed that the accuracy of structure-factor amplitudes is more important for refinement than for phasing, so in practice this restriction may not limit the application of our method or other methods that require reasonably accurate 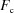
’s. However, it may be possible to infer the required error parameters even in the absence of an initial model, using intensity statistics such as the Wilson plot. But a brute-force strategy may provide an even better alternative, since the corrections require only a few parameters. In that case, parameters within a reasonable range could be tried, generating corrected data for phasing. In marginal cases where dynamical scattering prevents initial phasing, such an approach could be helpful. The method could be further improved by implementation at the stage of data integration and/or scaling, using the fact that only reflections simultaneously in the Bragg condition for that particular frame can be affected.

Electron crystallography can produce accurate atomic models that conform to expected bonding geometries to a remarkably high degree. Yet, using current methods, X-ray diffraction models fit better to the experimental data, compared with models refined against electron diffraction data. As X-ray data are obtained from crystals that have many million times more molecules, this observation should not be too surprising. One of those components that compromises the fit between model and data is dynamical electron scattering. Here we demonstrated that experimental approaches aimed at reducing crystal size and computational reduction of the effects of dynamical scattering lead to improved refinement statistics and model geometry. As the two approaches are independent, their combined effects are multiplied, leading to structures with improved geometry that are less biased by prior assumptions and fit better to the observed diffraction data.

## Supplementary Material

Crystal structure: contains datablock(s) IRELOH, EPICZA. DOI: 10.1107/S2053273318013918/td5055sup1.cif

Structure factors: contains datablock(s) IRELOH. DOI: 10.1107/S2053273318013918/td5055IRELOHsup2.hkl

Structure factors: contains datablock(s) EPICZA. DOI: 10.1107/S2053273318013918/td5055EPICZAsup3.hkl

Supporting figures and tables to the main text. DOI: 10.1107/S2053273318013918/td5055sup4.pdf

Lysozyme electron diffraction data (Timepix, CBF format) URL: https://doi.org/10.5281/zenodo.1250447

Electron diffraction data for IRELOH and EPICZA URL: https://doi.org/10.5281/zenodo.1407682

CCDC references: 1870980, 1870981

## Figures and Tables

**Table 1:** Data-merging statistics Reference values of unit-cell dimensions for IRELOH were obtained from Dai *et al.* (2010 ▸) and from Deffieu *et al.* (1977 ▸) for EPICZA. Values in parentheses correspond to the highest resolution shell, and data were truncated at approximately *I*/σ(*I*) > 1.0 and *CC*_1/2_ > 50% (Karplus & Diederichs, 2012 ▸; Diederichs & Karplus, 2013 ▸), see also Fig. S4 in the supporting information.

	IRELOH	EPICZA
Literature		
Chemical formula	C_16_H_18_O_5_	C_18_H_16_N_2_O_6_S_2_·H_2_O
Space group	*P*2_1_2_1_2_1_	*P*2_1_2_1_2_1_
Unit-cell dimensions		
*a*, *b*, *c* (Å)	8.06, 10.00, 17.73	11.11, 12.61, 13.49
α, β, γ (°)	90.00, 90.00, 90.00	90.00, 90.00, 90.00
		
Data integration		
Number of crystals†	3	4
Space group	*P*2_1_2_1_2_1_	*P*2_1_2_1_2_1_
Unit-cell dimensions		
*a*, *b*, *c* (Å)	8.090 (2), 9.940 (2), 17.700 (4)	11.080 (2), 12.580 (2), 13.440 (3)
α, β, γ (°)	90.00, 90.00, 90.00	90.00, 90.00, 90.00
Resolution (Å)	17.60–0.82 (0.85–0.82)	12.63–0.83 (0.85–0.83)
*I*/σ(*I*)	6.47 (2.50)	5.93 (1.45)
*CC*_1/2_ (%)	98.2 (46.2)	98.6 (67.7)
*R*_merge_ (%)	16.6 (50.0)	20.6 (28.6)
*R*_meas_ (%)	18.5 (61.2)	22.2 (39.8)
Completeness (%)	84.6 (78.9)	89.6 (36.4)
Reflections	6096 (352)	12039 (70)
Unique observations	1332 (135)	1761 (59)

† See Tables S2 and S3 in the supporting information for the data-processing statistics of the individual crystal data sets.

**Table 2:** Model building and refinement statistics without refining hydrogen atoms

	IRELOH	EPICZA
Space group	*P*2_1_2_1_2_1_	*P*2_1_2_1_2_1_
Unit-cell dimensions†		
*a*, *b*, *c* (Å)	8.015 (2), 10.015 (2), 17.703 (4)	10.996 (2), 12.452 (2), 13.218 (3)
α, β, γ (°)	90.00, 90.00, 90.00	90.00, 90.00, 90.00
Parameters	196	259
Restraints‡	156	267
Reflections	6096	12039
Unique	1332	1761
		
Refinement		
*R*1 (%)§	16.8 (15.0)	17.2 (15.4)
*R*_complete_ (%)¶	19.7	21.0
*w* *R*2 (%)	36.9	39.0
GooF	1.100	1.109
R.m.s.d. bond lengths (Å)††	0.022 (18)	0.027 (19)
		
Dynamical corrections		
*R*1 (%)§	13.2 (12.2)	12.7 (11.9)
*R*_complete_ (%)¶	15.1	14.3
*w* *R*2 (%)	29.5	29.1
GooF	0.944	0.879
R.m.s.d. bond lengths (Å)††	0.022 (17)	0.025 (13)

† New unit-cell dimensions after lattice refinement, see also Tables S9 and S10 in the supporting information.

‡ Enhanced rigid-bond restraints (RIGU) were applied for refinement in *SHELXL* (Thorn *et al.*, 2012 ▸).

§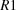 = 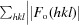 − 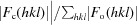 where the sum is over all reflections, values in parentheses show *R*1 for reflections 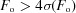.

¶ *R*_complete_ was calculated over all reflections with a 0.2% test set size as a robust and unbiased validation tool (Luebben & Gruene, 2015 ▸); since all data are included, *R*_work_ is equivalent to *R*1.

†† R.m.s.d. for all non-hydrogen atoms, calculated against reference values from high-resolution X-ray models (Dai *et al.*, 2010 ▸; Deffieu *et al.*, 1977 ▸); see also Tables S5 and S7 in the supporting information.

**Table 3:** Model building and refinement statistics after unconstrained refinement of hydrogen atoms

	IRELOH	EPICZA
Space group	*P*2_1_2_1_2_1_	*P*2_1_2_1_2_1_
Unit-cell dimensions†		
*a*, *b*, *c* (Å)	8.015 (2), 10.015 (2), 17.703 (4)	10.996 (2), 12.452 (2), 13.218 (3)
α, β, γ (°)	90.00, 90.00, 90.00	90.00, 90.00, 90.00
Parameters	245	305
Restraints‡	156	267
Reflections	6096	12039
Unique	1332	1761
		
Refinement		
*R*1 (%)§	15.7 (13.9)	16.6 (14.7)
*R*_complete_ (%)[Table-fn tfn10]	19.9	21.5
*w* *R*2 (%)	34.6	37.1
GooF	1.031	1.051
R.m.s.d. bond lengths (Å)[Table-fn tfn11]	0.024 (18)	0.030 (20)
R.m.s.d. hydrogen-bond lengths (Å)[Table-fn tfn12]	0.180 (72)	0.259 (80)
		
Dynamical corrections		
*R*1 (%)§	12.5 (11.5)	12.2 (11.4)
*R*_complete_ (%)[Table-fn tfn10]	15.2	14.5
*w* *R*2 (%)	28.1	28.0
GooF	0.907	0.851
R.m.s.d. bond lengths (Å)[Table-fn tfn11]	0.022 (13)	0.026 (13)
R.m.s.d. hydrogen-bond lengths (Å)[Table-fn tfn12]	0.073 (52)	0.110 (56)

† New unit-cell dimensions after lattice refinement, see also Tables S9 and S10 in the supporting information.

‡ Enhanced rigid-bond restraints (RIGU) were applied for refinement in *SHELXL* (Thorn *et al.*, 2012 ▸).

§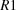 = 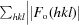 − 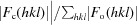 where the sum is over all reflections, values in parentheses show *R*1 for reflections 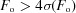.

¶ *R*_complete_ was calculated over all reflections with a 0.2% test set size as a robust and unbiased validation tool (Luebben & Gruene, 2015 ▸); since all data are included, *R*_work_ is equivalent to *R*1.

†† R.m.s.d. for all non-hydrogen atoms, calculated against reference values from high-resolution X-ray models (Dai *et al.*, 2010 ▸; Deffieu *et al.*, 1977 ▸).

‡‡ R.m.s.d. for the idealized hydrogen-bond lengths after unconstrained refinement of the hydrogen positions (Gruene *et al.*, 2014 ▸; Sheldrick, 2015*a* ▸); see also Tables S6 and S8 in the supporting information.

**Table 4:** Refinement statistics and dynamical scattering corrections of low-resolution protein data from seven lysozyme data sets recorded and solved previously (Clabbers *et al.*, 2017 ▸) Experimental data are available online at https://doi.org/10.5281/zenodo.1250447. Values in parentheses correspond to the highest resolution shell, and the data were truncated at approximately *I*/σ(*I*) > 1.0 and *CC*_1/2_ > 50% (Diederichs & Karplus, 2013 ▸).

	Lysozyme
Data integration	
Space group	*P*2_1_2_1_2
Unit-cell dimensions	
*a*, *b*, *c* (Å)	104.56, 68.05, 32.05
α, β, γ (°)	90.0, 90.0, 90.0
Number of crystals	7
Resolution (Å)	57.03–2.11 (2.17–2.11)
*R*_merge_ (%)	42.1 (57.2)
*CC*_1/2_ (%)	90.4 (60.3)
*I*/σ(*I*)	2.7 (1.0)
Completeness (%)	62.1 (49.8)
Reflections	41191 (1462)
Unique reflections	8560 (545)
	
Refinement	
Reflections	8503
*R*1 (%) †	24.4
*R*_complete_ (%) ‡	29.1
〈*B*〉 (Å^2^)	33.02
R.m.s.d. bond lengths (Å)	0.074
R.m.s.d. bond angles (°)	1.0706
Ramachandran	
Favoured, allowed, outliers (%)	98.4, 1.6, 0.0
	
Dynamical corrections	
Reflections	8503
*R*1 (%)†	24.3
*R*_complete_ (%)‡	26.2
〈*B*〉 (Å^2^)	41.09
R.m.s.d. bond lengths (Å)	0.066
R.m.s.d. bond angles (°)	1.0072
Ramachandran	
Favoured, allowed, outliers (%)	98.0, 2.0 0.0

†
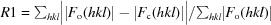

‡ We present *R*1 and *R*_complete_ instead of *R*_work_ and *R*_free_. With less than 10 000 unique reflections *R*_complete_ is preferred over *R*_free_ since it is calculated from all reflections (Brünger, 1997 ▸; Luebben & Gruene, 2015 ▸). Since all structure factors are used in turn, this leads to a more robust calculation than *R*_free._ With this validation method, the actual refinement uses all reflections, hence *R*_work_ is equivalent to *R*1.
