# Supplementary material for: Reducing dynamical electron scattering reveals hydrogen atoms
Source: Acta Crystallogr A Found Adv. 2019 Jan 1;75(Pt 1):82–93. doi: 10.1107/S2053273318013918 (PMC6302931; doi:10.1107/S2053273318013918)
Supplement: Supplementary file 4 [file a-75-00082-sup4.pdf]

Acta Cryst A
FOUNDATIONS
ADVANCES

Volume 75 (2019)

Supporting information for article:

Reducing dynamical electron scattering reveals hydrogen atoms

Max T. B. Clabbers, Tim Gruene, Eric van Genderen and Jan Pieter Abrahams

Reducing dynamical electron scattering reveals hydrogen atoms

Supporting information

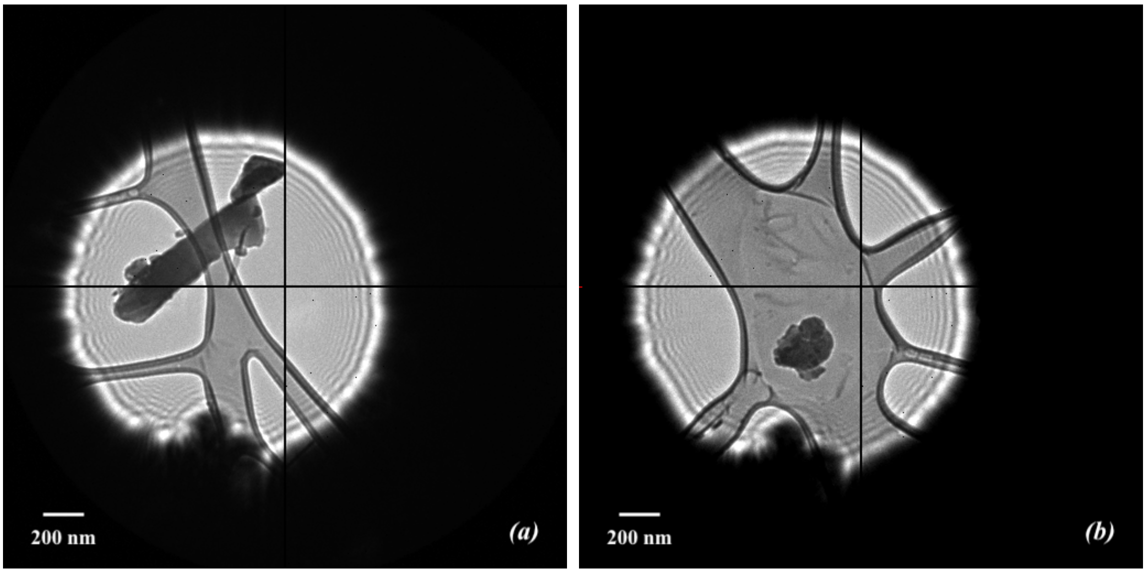

Figure S1. Micrographs of a 900 by 200 nm IRELOH crystal (a), and a 300 by 200 nm EPICZA crystal (b). Diffraction data were collected with a ~2.0 μm diameter parallel beam that was also used for acquiring the images on the Timepix detector, following directly after diffraction data acquisition.

Table S2. Data processing statistics of three individual IRELOH crystals

	1	2	3
Data acquisition			
$\Delta\varphi_{frame}$[1] [°]	0.0652	0.0652	0.0652
φ_{total}[2] [°]	56.46	48.90	38.50
Detector distance[3] [mm]	489	489	489
Data integration			
Space group	$P2_12_12_1$	$P2_12_12_1$	$P2_12_12_1$
Unit cell dimensions			
a, b, c [Å]	8.28(5), 9.91(6), 17.84(8)	8.09(3), 9.94(8), 17.70(5)	8.06(4), 10.27(7), 17.59(5)
α, β, γ [°]	90.00, 90.00, 90.00	90.00, 90.00, 90.00	90.00, 90.00, 90.00
Resolution [Å][4]	9.91-1.01 (1.07-1.01)	6.61-0.80 (0.85-0.80)	17.60-0.80 (0.85-0.80)
I/σI	5.03 (1.07)	5.86 (1.48)	4.94 (1.33)
$CC_{1/2}$ [%]	99.6 (77.9)	97.9 (37.9)	98.2 (45.7)
R_{merge} [%]	13.6 (57.1)	11.5 (51.1)	12.0 (51.4)
R_{meas} [%]	16.6 (72.7)	14.3 (67.6)	14.8 (63.8)
Completeness [%]	53.2 (52.8)	60.03 (59.0)	49.8 (35.7)
Reflections	1278 (141)	2734 (269)	2252 (185)
Unique observations	481 (75)	1004 (148)	860 (95)

[1] Angular per frame increment during data acquisition, defined as the total tilt range φ_{total} divided by the number of frames (data were acquired with an exposure time of 0.1 s).

[2] The total rotation range over which data were acquired.

[3] The path length between the sample and the detector, the detector distance was calibrated using an aluminum diffraction standard and was not refined during data processing.

[4] Values in parentheses correspond to the highest resolution shell, data were truncated at approximately I/σI > 1.0 and $CC_{1/2}$ > 50% where the correlation is still significant (Karplus & Diederichs, 2012; Diederichs & Karplus, 2013).

Table S3. Data processing statistics of four individual EPICZA crystals

	1	2	3	4
Data acquisition				
$\Delta\varphi_{frame}$[5] [°]	0.0662	0.0664	0.0664	0.0710
φ_{total}[6] [°]	38.33	57.78	57.78	58.93
Detector distance[7] [mm]	489	489	489	489
Data integration				
Space group	$P2_12_12_1$	$P2_12_12_1$	$P2_12_12_1$	$P2_12_12_1$
Unit cell dimensions				
a, b, c [Å]	11.08(4), 12.58(2), 13.44(1)	11.07(7), 12.63(7), 13.34(9)	11.02(10), 12.78(5), 13.33(1)	11.07(1), 12.13(3), 13.63(2)
α, β, γ [°]	90.00, 90.00, 90.00	90.00, 90.00, 90.00	90.00, 90.00, 90.00	90.00, 90.00, 90.00
Resolution [Å][8]	12.58-0.86 (0.91-0.86)	12.63-0.82 (0.87-0.82)	8.35-0.88 (0.94-0.88)	11.07-0.90 (0.95-0.90)
I/σI	6.57 (1.21)	6.02 (1.54)	4.90 (1.07)	5.93 (1.57)
$CC_{1/2}$ [%]	99.2 (70.8)	99.1 (73.2)	99.2 (53.2)	99.0 (41.9)
R_{merge} [%]	9.7 (41.0)	9.5 (22.9)	11.5 (49.3)	14.2 (51.1)
R_{meas} [%]	12.1 (51.2)	12.0 (32.3)	14.0 (63.8)	16.5 (59.3)
Completeness [%]	47.4 (49.3)	68.5 (41.1)	71.0 (68.7)	54.0 (41.5)
Reflections	2198 (226)	3513 (169)	3291 (398)	3068 (315)
Unique observations	835 (135)	1370 (127)	1173 (178)	824 (97)

5 Angular per frame increment during data acquisition, defined as the total tilt range φ_{total} divided by the number of frames (data were acquired with an exposure time of 0.1 s).

6 The total rotation range over which data were acquired.

7 The path length between the sample and the detector, the detector distance was calibrated using an aluminum diffraction standard and was not refined during data processing.

8 Values in parentheses correspond to the highest resolution shell, data were truncated at approximately I/σI > 1.0 and $CC_{1/2}$ > 50% if the correlation is still significant (Karplus & Diederichs, 2012; Diederichs & Karplus, 2013).

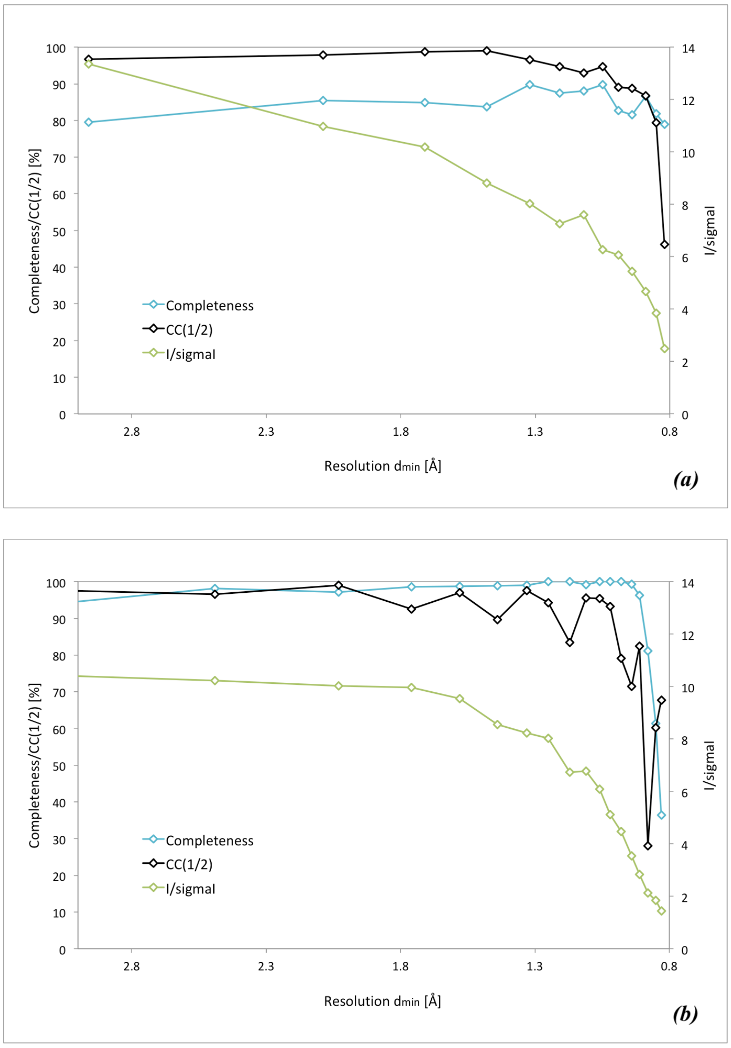

Figure S4. Merging statistics for IRELOH (a) and EPICZA (b), data completeness and quality indicators $CC_{1/2}$ and I/σI are plotted as function of the resolution d_{min}. Data were integrated, scaled and merged using *XDS* (Kabsch, 2010). Data were truncated at approximately I/σI > 1.0 and $CC_{1/2}$ > 50% (Karplus & Diederichs, 2012; Diederichs & Karplus, 2013).

Table S5. Bond length comparison for IRELOH between the X-ray model, the electron diffraction model, and the electron diffraction model after applying the dynamical corrections

		X-ray[9]	ED	ED corrected
		d_X [Å]	d_e [Å]	d_e [Å]
C16	C15	1.513(4)	1.47(2)	1.475(15)
C15	C14	1.506(3)	1.523(22)	1.534(13)
C14	C13	1.542(4)	1.54(2)	1.539(15)
C13	C12	1.526(4)	1.538(18)	1.536(13)
C12	C11	1.490(3)	1.493(16)	1.481(10)
C11	C10	1.328(3)	1.348(17)	1.356(10)
C10	C9	1.441(3)	1.476(16)	1.470(11)
C9	O9	1.232(3)	1.201(18)	1.205(12)
C9	C8	1.511(3)	1.503(19)	1.506(13)
C8	C3	1.389(3)	1.445(18)	1.446(13)
C3	C2	1.506(3)	1.505(18)	1.498(12)
C2	C1	1.501(4)	1.517(18)	1.513(12)
C1	O1	1.193(3)	1.210(19)	1.216(13)
C1	O16	1.319(3)	1.330(18)	1.329(12)
C15	O16	1.467(3)	1.461(16)	1.466(11)
C3	C4	1.402(3)	1.385(19)	1.389(14)
C4	C5	1.372(3)	1.350(18)	1.357(12)
C5	O5	1.364(3)	1.382(19)	1.370(13)
C5	C6	1.395(3)	1.41(2)	1.419(14)
C6	C7	1.376(3)	1.36(2)	1.345(15)
C7	C8	1.391(3)	1.393(17)	1.402(11)
C7	O7	1.369(3)	1.360(17)	1.363(12)
			rmsd [Å] [10]	rmsd [Å] [10]
			0.022(18)	0.022(17)

[9] Reference bond lengths for equivalent bonds to the electron diffraction model were taken from a previously reported X-ray model (Dai *et al.*, 2010)

[10] $rmsd = \sqrt{\sum_{i=1}^{N}(d_{X,i} - d_{e,i})^2/N}$

Table S6. Hydrogen bond lengths after unconstrained refinement of the hydrogen positions for the electron diffraction model and the electron diffraction model after applying the dynamical corrections

			ED	ED corrected
		dX-H[11]	d_e [Å]	d_e [Å]
C2	H2A	1.090	1.206(75)	1.085(55)
C2	H2B	1.090	1.066(71)	0.981(51)
C4	H4	1.080	1.091(65)	1.096(49)
O5	H5	0.994	0.906(78)	0.922(56)
C6	H6	1.080	1.112(68)	1.099(47)
O7	H7	1.048	1.025(75)	1.034(54)
C10	H10	1.080	1.258(73)	1.040(52)
C11	H11	1.080	0.929(71)	0.943(53)
C12	H12A	1.090	0.955(75)	0.982(54)
C12	H12B	1.090	0.937(82)	1.088(58)
C13	H13A	1.090	1.132(70)	1.075(46)
C13	H13B	1.090	1.445(68)	1.103(52)
C14	H14A	1.090	1.293(62)	1.178(45)
C14	H14B	1.090	1.186(73)	1.133(53)
C15	H15	1.100	1.282(70)	1.213(52)
C16	H16A	1.060	1.475(75)	1.180(56)
C16	H16B	1.060	1.296(73)	1.148(57)
C16	H16C	1.060	1.218(67)	1.084(51)
			rmsd [Å] [12]	rmsd [Å] [12]
			0.180(72)	0.073(52)

[11] Idealised hydrogen bond lengths were generated in *SHELXL* using the NEUT command (Gruene *et al.*, 2014; Sheldrick, 2015)

[12] $rmsd = \sqrt{\sum_{i=1}^{N}(d_{X-H,i} - d_{e,i})^2/N}$

Table S7. Bond length comparison for EPICZA between the X-ray model, the electron diffraction model, and the electron diffraction model after applying the dynamical corrections

		X-ray[13]	ED	ED corrected
		d_X [Å]	d_e [Å]	d_e [Å]
S1	S2	2.083(1)	2.096(11)	2.099(7)
S1	C1	1.873(3)	1.864(16)	1.863(10)
S2	C3	1.863(3)	1.876(17)	1.876(11)
C1	N1	1.450(4)	1.409(19)	1.414(12)
N1	C2	1.337(4)	1.39(2)	1.387(13)
C2	C3	1.529(4)	1.529(19)	1.529(12)
C3	N2	1.455(4)	1.422(19)	1.429(12)
N2	C4	1.335(4)	1.33(2)	1.322(12)
C4	C1	1.526(4)	1.554(19)	1.547(11)
N2	C12	1.471(4)	1.468(17)	1.462(11)
C12	C13	1.529(5)	1.55(2)	1.550(14)
C13	C14	1.512(5)	1.47(2)	1.496(12)
C14	C3	1.522(5)	1.54(2)	1.527(13)
C12	C18	1.523(4)	1.540(19)	1.523(12)
C18	C17	1.504(5)	1.48(2)	1.489(13)
C17	C16	1.344(6)	1.334(25)	1.344(15)
C16	C15	1.458(6)	1.47(2)	1.472(13)
C15	C13	1.527(5)	1.52(2)	1.520(13)
C18	O5	1.425(4)	1.41(2)	1.389(14)
C15	O6	1.215(5)	1.22(2)	1.218(14)
C1	C5	1.529(4)	1.53(2)	1.533(13)
C5	C6	1.527(4)	1.48(2)	1.479(13)
C6	C7	1.536(4)	1.53(2)	1.530(14)
C7	N1	1.481(4)	1.504(19)	1.492(12)
C2	O2	1.235(4)	1.22(2)	1.225(13)
C4	O1	1.227(4)	1.26(2)	1.267(13)
C6	C8	1.496(4)	1.52(2)	1.531(14)
C8	C9	1.491(5)	1.53(2)	1.512(15)
C9	C10	1.323(5)	1.28(3)	1.293(17)
C10	C11	1.512(5)	1.53(2)	1.511(15)
C11	C7	1.511(4)	1.54(2)	1.541(14)
C11	O3	1.429(4)	1.39(2)	1.397(13)
C8	O4	1.221(4)	1.17(2)	1.159(14)
			rmsd [Å] [14]	rmsd [Å] [14]
			0.027(19)	0.025(13)

[13] Reference bond lengths for equivalent bonds to the electron diffraction model were taken from a previously reported X-ray model (Deffieux *et al.*, 1977).

[14] $rmsd = \sqrt{\sum_{i=1}^{N}(d_{X,i} - d_{e,i})^2/N}$

Table S8. Hydrogen bond lengths after unconstrained refinement of the hydrogen positions for the electron diffraction model and the electron diffraction model after applying the dynamical corrections

			ED	ED corrected
		dX-H[15]	d_e [Å]	d_e [Å]
O3	H3	0.980	0.900(56)	0.900(42)
C5	H5A	1.090	1.244(81)	1.116(52)
C5	H5B	1.090	1.232(80)	1.106(48)
O5	H5	0.980	1.021(13)	1.050(64)
C7	H7	1.100	1.626(84)	1.184(54)
C6	H6	1.100	0.950(84)	1.118(52)
C9	H9	1.080	1.434(94)	1.189(60)
C10	H10	1.080	1.680(106)	1.154(66)
C11	H11	1.100	1.193(96)	1.128(57)
C12	H12	1.100	1.103(73)	1.065(46)
C13	H13	1.100	1.303(85)	1.178(51)
C14	H14A	1.090	0.910(87)	1.000(69)
C14	H14B	1.090	1.223(97)	1.154(72)
C16	H16	1.080	1.429(87)	1.170(51)
C17	H17	1.080	1.211(80)	1.119(51)
C18	H18	1.100	1.117(87)	0.744(54)
			rmsd [Å] [16]	rmsd [Å] [16]
			0.259(80)	0.110(56)

[15] Idealised hydrogen bond lengths were generated in *SHELXL* using the NEUT command (Gruene *et al.*, 2014; Sheldrick, 2015)

[16] $rmsd = \sqrt{\sum_{i=1}^{N}(d_{X-H,i} - d_{e,i})^2/N}$

Table S9. Restraints on 1,2-distances (DFIX) and 1,3-distances (DANG) used by *SHELXL* and *CellOpt*[17] for lattice refinement of IRELOH, idealised geometrical restraints were generated using the *Grade* web server.

```
DFIX 1.508 0.016 C16 C15
DFIX 1.518 0.021 C15 C14
DFIX 1.524 0.019 C14 C13
DFIX 1.527 0.024 C13 C12
DFIX 1.497 0.024 C12 C11
DFIX 1.319 0.017 C11 C10
DFIX 1.470 0.015 C10 C9
DFIX 1.238 0.018 C9 O9
DFIX 1.494 0.022 C9 C8
DFIX 1.408 0.013 C8 C3
DFIX 1.512 0.008 C3 C2
DFIX 1.504 0.009 C2 C1
DFIX 1.202 0.015 C1 O1
DFIX 1.341 0.016 C1 O16
DFIX 1.466 0.010 C15 O16
DFIX 1.391 0.010 C3 C4
DFIX 1.387 0.009 C4 C5
DFIX 1.365 0.014 C5 O5
DFIX 1.387 0.009 C5 C6
DFIX 1.385 0.010 C6 C7
DFIX 1.410 0.013 C7 C8
DFIX 1.358 0.015 C7 O7
```

```
DANG 2.245 0.022 O1 O16
DANG 2.406 0.025 C2 O1
DANG 2.351 0.026 C2 O16
DANG 2.404 0.022 C1 C15
DANG 2.405 0.033 C16 O16
DANG 2.396 0.036 C14 O16
DANG 2.535 0.033 C16 C14
DANG 2.524 0.029 C1 C3
DANG 2.506 0.024 C2 C4
DANG 2.563 0.024 C2 C8
DANG 2.419 0.020 C4 C8
DANG 2.417 0.016 C3 C5
DANG 2.381 0.033 C4 O5
DANG 2.411 0.015 C4 C6
DANG 2.381 0.033 C6 O5
DANG 2.397 0.014 C5 C7
DANG 2.372 0.036 C6 O7
DANG 2.431 0.019 C6 C8
DANG 2.412 0.027 C8 O7
DANG 2.499 0.037 C7 C9
DANG 2.419 0.019 C7 C3
DANG 2.536 0.033 C3 C9
DANG 2.364 0.028 C8 O9
DANG 2.576 0.042 C10 C8
DANG 2.359 0.027 C10 O9
DANG 2.451 0.037 C11 C9
DANG 2.511 0.031 C10 C12
DANG 2.520 0.048 C11 C13
DANG 2.562 0.031 C12 C14
DANG 2.567 0.038 C13 C15
```

[17] https://github.com/JLuebben/CellOpt

Table S10. Restraints on 1,2-distances (DFIX) and 1,3-distances (DANG) used by *SHELXL* and *CellOpt*[18] for lattice refinement of EPICZA, idealised geometrical restraints were generated using the *Grade* web server.

```
DFIX 1.429 0.011 C11 O3
DFIX 1.499 0.010 C11 C10
DFIX 1.326 0.011 C10 C9
DFIX 1.464 0.013 C9 C8
DFIX 1.222 0.012 C8 O4
DFIX 1.507 0.020 C8 C6
DFIX 1.537 0.012 C6 C5
DFIX 1.530 0.012 C5 C1
DFIX 1.876 0.015 C1 S1
DFIX 2.059 0.026 S1 S2
DFIX 1.876 0.015 C3 S2
DFIX 1.530 0.012 C3 C14
DFIX 1.537 0.012 C14 C13
DFIX 1.546 0.017 C13 C12
DFIX 1.525 0.009 C12 C18
DFIX 1.429 0.011 C18 O5
DFIX 1.499 0.010 C18 C17
DFIX 1.326 0.011 C17 C16
DFIX 1.464 0.013 C16 C15
DFIX 1.507 0.020 C15 C13
DFIX 1.222 0.012 C15 O6
DFIX 1.479 0.011 C12 N2
DFIX 1.456 0.016 C3 N2
DFIX 1.348 0.011 C4 N2
DFIX 1.520 0.011 C4 C1
DFIX 1.216 0.012 C4 O1
DFIX 1.520 0.011 C3 C2
DFIX 1.216 0.012 C2 O2
DFIX 1.348 0.011 C2 N1
DFIX 1.456 0.016 C1 N1
DFIX 1.479 0.011 C7 N1
DFIX 1.525 0.009 C7 C11
DFIX 1.546 0.017 C7 C6
```

```
DANG 2.258 0.020 N1 O2
DANG 2.381 0.023 C3 O2
DANG 2.426 0.033 C3 N1
DANG 2.472 0.036 C2 C1
DANG 2.486 0.034 C2 C11
DANG 2.413 0.032 C1 C7
DANG 2.332 0.026 C5 N1
DANG 2.761 0.025 N1 S1
DANG 2.483 0.026 C4 N1
DANG 2.672 0.031 C4 S1
DANG 2.851 0.051 C5 S1
DANG 2.557 0.028 C4 C5
DANG 2.385 0.030 C1 C6
DANG 2.364 0.022 C6 N1
DANG 2.476 0.046 C11 N1
DANG 2.545 0.045 C11 C6
DANG 2.556 0.057 C7 C8
DANG 2.433 0.020 C7 C5
DANG 2.579 0.071 C5 C8
DANG 2.390 0.026 C6 O4
DANG 2.520 0.036 C6 C9
DANG 2.347 0.024 C9 O4
DANG 2.432 0.021 C8 C10
DANG 2.479 0.023 C11 C9
DANG 2.457 0.026 C7 O3
DANG 2.467 0.038 C7 C10
DANG 2.397 0.037 C10 O3
DANG 2.672 0.031 C2 S2
DANG 2.483 0.026 C2 N2
DANG 2.557 0.028 C2 C14
DANG 2.761 0.025 N2 S2
DANG 2.855 0.051 C14 S2
DANG 2.332 0.026 C14 N2
DANG 2.965 0.026 C3 S1
DANG 2.965 0.026 C1 S2
DANG 2.472 0.036 C3 C4
DANG 2.413 0.032 C3 C12
DANG 2.486 0.034 C4 C12
DANG 2.258 0.020 N2 O1
DANG 2.426 0.033 C1 N2
DANG 2.381 0.023 C1 O1
DANG 2.459 0.047 C18 N2
DANG 2.364 0.022 C13 N2
DANG 2.545 0.045 C18 C13
DANG 2.457 0.026 C12 O5
DANG 2.467 0.038 C12 C17
DANG 2.397 0.037 C17 O5
DANG 2.479 0.023 C16 C18
DANG 2.432 0.021 C15 C17
DANG 2.347 0.024 C16 O6
DANG 2.520 0.036 C13 C16
DANG 2.390 0.026 C13 O6
DANG 2.579 0.071 C15 C14
DANG 2.556 0.057 C15 C12
DANG 2.433 0.020 C12 C14
DANG 2.385 0.030 C3 C13
```

[18] https://github.com/JLuebben/CellOpt

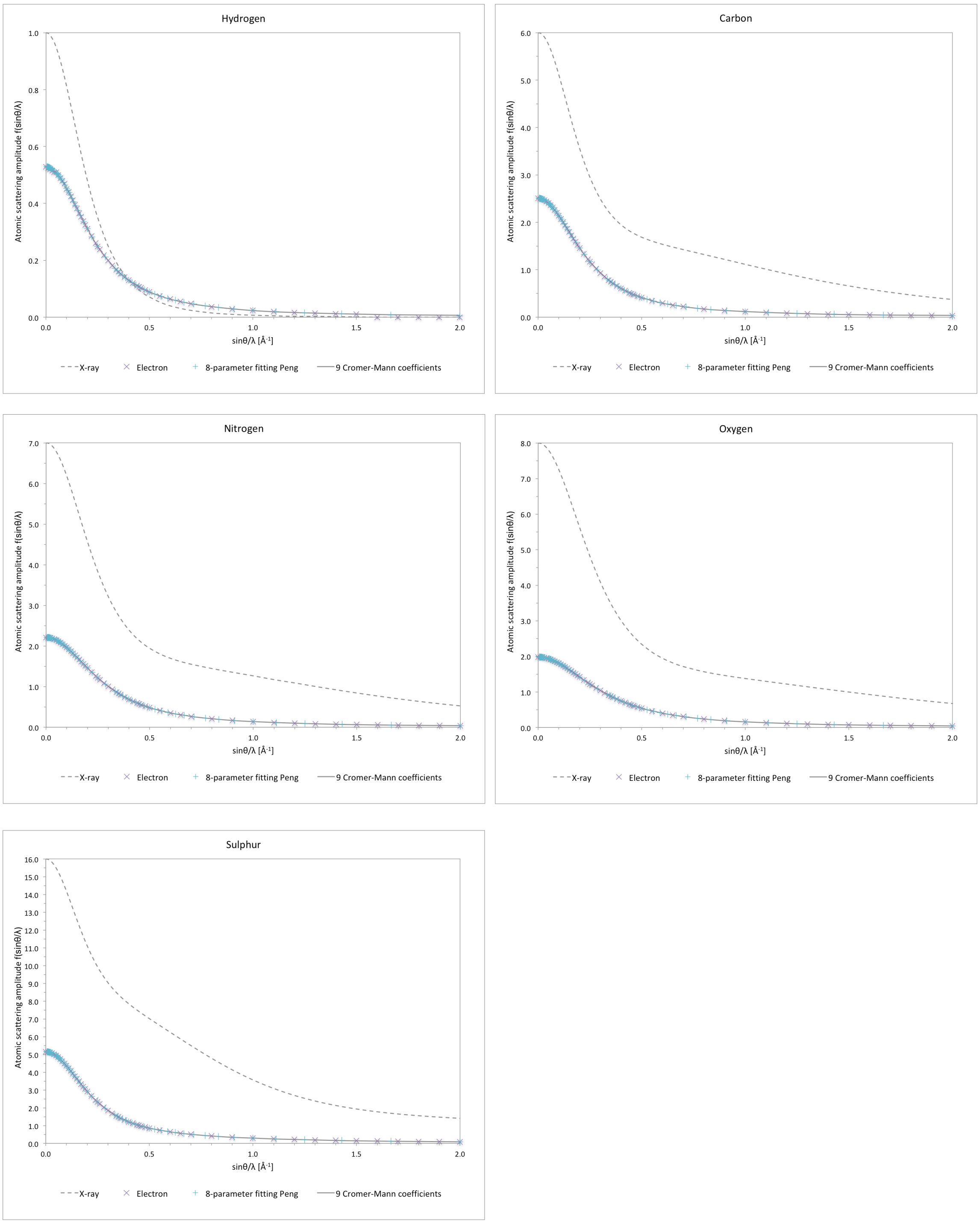

Figure S11. Atomic scattering factors plotted as function of $sin\theta/\lambda$. Tabulated values for the mean atomic scattering factors for X-ray[19] and electron[20] diffraction were taken from literature. Values for the 8-parameter fitting for electron scattering factors were taken from Peng, 1999. The atomic electron scattering factors used for modeling and refinement in *SHELXL* are calculated from 9 Cromer-Mann coefficients with the expansion[21] $f(\sin\theta/\lambda) = \sum_{i=1}^{4} a_i\, e^{(-b_i sin^2\theta/\lambda^2)} + c$. The 9 Cromer-Mann coefficients were fitted to the 8-parameter fitting from Peng, 1999. Plotting of the curve for the 9 Cromer-Mann coefficients confirms a consistent fit of the scattering factors to the literature values.

---

[19] Table 6.1.1.1, *International Tables for Crystallography* (2006), Vol. C, Section 6.1, pp. 555-564

[20] Table 4.3.1.1, *International Tables for Crystallography* (2006), Vol. C, Section 4.3, pp. 263-271

[21] Equation 6.1.1.15, *International Tables for Crystallography* (2006), Vol. C, Section 6.1, p. 565

References

Dai, J., Krohn, K., Flörke, U., Pescitelli, G., Kerti, G., Papp, T., Kövér, K. E., Bényei, A. C., Draeger, S., Schulz, B. & Kurtán, T. (2010). *European J. Org. Chem.* **2010**, 6928–6937.

Deffieux, G., Gadret, M., Leger, J. M. & Carpy, A. (1977). *Acta Crystallogr. Sect. B*. **33**, 1474–1478.

Diederichs, K. & Karplus, P. A. (2013). *Acta Cryst. D*. **69**, 1215–1222.

Gruene, T., Hahn, H. W., Luebben, A. V., Meilleur, F. & Sheldrick, G. M. (2014). *J. Appl. Crystallogr.* **47**, 462–466.

Kabsch, W. (2010). *Acta Cryst. D*. **66**, 125–132.

Karplus, P. A. & Diederichs, K. (2012). *Science (80-.).* **336**, 1030–1033.

Peng, L. M. (1999). *Micron*. **30**, 625–648.

Sheldrick, G. M. (2015). *Acta Cryst. C*. **71**, 3–8.
